# Breastfeeding exclusivity and duration: trends and inequalities in four population-based birth cohorts in Pelotas, Brazil, 1982–2015

**DOI:** 10.1093/ije/dyy159

**Published:** 2019-03-18

**Authors:** Iná S Santos, Fernando C Barros, Bernardo L Horta, Ana M B Menezes, Diego Bassani, Luciana Tovo-Rodrigues, Natália P Lima, Cesar G Victora, Pelotas Cohorts Study Group, Aluisio J D Barros, Alicia Matijasevich, Andrea Dâmaso Bertoldi, Fernando C Wehrmeister, Helen Gonçalves, Joseph Murray, Maria Cecilia F Assunção, Mariangela F Silveira, Marlos Rodrigues Domingues, Pedro R C Hallal

**Affiliations:** 1 Federal University of Pelotas, Brazil; 2 University of São Paulo, Brazil; 1 Post Graduate Program in Epidemiology, Federal University of Pelotas, Pelotas, Brazil; 2 Post-Graduate Program in Health and Behavior, Catholic University of Pelotas, Pelotas, Brazil; 3 Department of Pediatrics, Hospital for Sick Children, University of Toronto, Toronto, ON, Canada

**Keywords:** Breast feeding, exclusive breastfeeding, socioeconomic factors, cohort studies

## Abstract

**Background:**

Brazil has made substantial improvements in the duration of breastfeeding. We use data from four population-based cohorts to examine how trends and inequalities in breastfeeding indicators changed over time in a Brazilian city.

**Methods:**

Data from four birth cohorts, each including all births in a calendar year (1982, 1993, 2004 and 2015) in the city of Pelotas were used. Information on breastfeeding was collected when children were aged between 3 and 20 months. The prevalences of continued breastfeeding at 1 year of age and of exclusive breastfeeding at 3 months were calculated according to family income, maternal skin colour and sex.

**Results:**

Prevalence of breastfeeding at 12 months increased from 16% to 41% in the 33-year period. The prevalence of exclusive breastfeeding at 3 months increased from 7% in 1993 to 45% in 2015. Increases in exclusive breastfeeding at 3 months were seen in all socioeconomic groups, but the 2015 rates remain highest (57.2%) among the women in the richest quintile, and lowest among those in the poorest quintile (34.6%). Black mothers were more likely to breastfeed at 12 months than Whites in the four cohorts. In the earlier cohorts, breastfeeding at 12 months was more common among the poor, but by 2015 these differences had disappeared.

**Conclusions:**

There were important positive changes in breastfeeding practices during this period, but less than half of the children in 2015 were receiving the full benefits of breast milk. Improved breastfeeding practices are being adopted by high-income women to a greater extent than by poor women.

**Key Messages**

- The prevalence of continued breastfeeding at 1 year in the 2015 Pelotas cohort (41%) was similar to the Brazilian national estimate in 2013 (45.4%). Prevalence of exclusive breastfeeding at 3 months of age was 45%.
- There were important positive changes in breastfeeding practices from 1982 to 2015, but less than half of the children in 2015 were receiving the full benefits of breast milk.
- Improved breastfeeding practices are being adopted by high-income women to a greater extent than by poor women. Promotion efforts should be reinforced for the latter group.

## Introduction

The short- and long-term benefits of breastfeeding for both mothers and children are well documented. In the short term, breastfeeding—and in particular exclusive breastfeeding—protects against infectious diseases, especially diarrhoea and pneumonia.^1–4^ In the long term, breastfeeding has been associated with lower risks of obesity and type 2 diabetes, increased intelligence in childhood, adolescence and adulthood and higher levels of formal education and income in adult life.^5^^,^^6^

In terms of maternal health, breastfeeding provides protection against breast cancer and contributes to increased birth spacing, while also potentially protecting against ovarian cancer and type 2 diabetes.^4^ More recently, breastmilk has been recognized as a living substance containing stem and progenitor cells as well as oligosaccharides that promote the growth of a healthy microbiome and also present anti-infective properties.^4^^,^^7^^,^^8^

In spite of the well recognized benefits of breastfeeding, rates of early initiation of breastfeeding and prevalence of exclusive breastfeeding among infants aged less than 5 months in most low- and middle-income countries remain below 50%.^9^ A previous analysis including the first three Pelotas birth cohorts (1982, 1993 and 2004) indicated an improvement in breastfeeding indicators. In those two decades, the proportion of 12-month-old children who were breastfed increased from 16% to 37% and the proportion of those exclusively breastfeeding at 3 months of age, which was null in 1982, reached 27% of the infants in 2004.^10^ The objective of the present study was to assess whether these positive trends persisted when the results of the Pelotas 2015 Birth Cohort were incorporated in the analyses.

## Methods

The four Pelotas Birth Cohorts (1982, 1993, 2004 and 2015) comprise about 20 000 participants. All newborns to mothers resident at the urban area of the municipality and delivered in all delivery wards in the city, between 1 January and 31 December of the corresponding year, were eligible to participate in the study. A total of 5914 newborns were enrolled in 1982, 5249 in 1993, 4231 in 2004 and 4275 in 2015, representing more than 99% of all births that occurred in Pelotas in those years.^11^

At the beginning of 1983, the 1982 cohort team tried to locate all the participants born between January and April 1982 (*n* = 1916), being able to locate 79.3% of that sample (mean age 11.3 months); the full cohort was re-visited in early 1984 through a census of all households in the city, when the follow-up rate increased to 87.2%. A sample of the 1993 cohort was followed up at 6 and 12 months (*n* = 1460). The sample included all low-birthweight newborns (<2500 g) and 20% of the remaining cohort members. At 6 and 12 months, 96.8% and 93.4%, respectively, of the intended samples were assessed. At 3 and 12 months of age, the cohort teams attempted to contact all infants that were part of the 2004 and 2015 birth cohorts. The follow-up rates in 2004 were 95.7% and 94.3%, respectively; and in 2015 were 97.2% and 95.4%. Supplementary Table 1, available as Supplementary data at *IJE* online, describes the sample size and timing of each follow-up for each cohort; more details are provided in the first article in this supplement.^11^

In the four cohorts, mothers were interviewed at the hospital in the first 24 h after the delivery and the newborns were examined with standardized techniques and data collection instruments. Information was collected from the mother on social, demographic and health-related variables.^11^ Home visits were carried at the ages described above, and information on the age when breastfeeding was stopped and on the introduction of complementary foods during first year of life was collected. Breastfeeding pattern in the first year of life was classified in four groups: exclusive breastfeeding (breastfed infants who did not receive any other fluids or solid foods); predominant breastfeeding (breastfed infants who received fluids such as water, tea or fruit juices, but were not fed solid or semi-solid foods); partial breastfeeding (infants who were fed breast milk complemented with other types of milk, such as cow’s milk or formula, or with solid or semi-solid foods); and weaned infants (who were not breastfed). In 1982, questions were not asked about the use of water or tea, so that it was not possible to estimate the prevalence of exclusive breastfeeding.

Information on monthly family income, maternal skin colour (white, brown or black) and child sex obtained in the perinatal study constituted the independent variables in the analyses. Family income was divided in quintiles, with the first quintile including the poorest and the fifth quintile the wealthiest families. Ethnic group classification according to self-reported skin colour has been officially adopted in Brazil, and is supported by the Organized Black Movement which since the 1970s has advocated for disaggregation of all vital and health statistics according to skin colour.^12^

For the analyses, data of the 1993 cohort were weighted in order to account for the oversampling of low-birthweight infants. The prevalences of exclusive breastfeeding at 3 months of age and of continued breastfeeding at 1 year of age, according to independent variables, were calculated for each cohort. Chi-square tests were used to assess the association between breastfeeding and the independent variables. When appropriate, chi-square tests for linear trends in proportions were used to assess differences over time.

The slope index of inequality (SII) and concentration index (CIX) were calculated for family income in quintiles, to assess absolute and relative inequalities in prevalence of exclusive breastfeeding at 3 months of age and in continued breastfeeding at 1 year of age in the four cohorts.^13^^,^^14^

## Results

**Figure 1:**
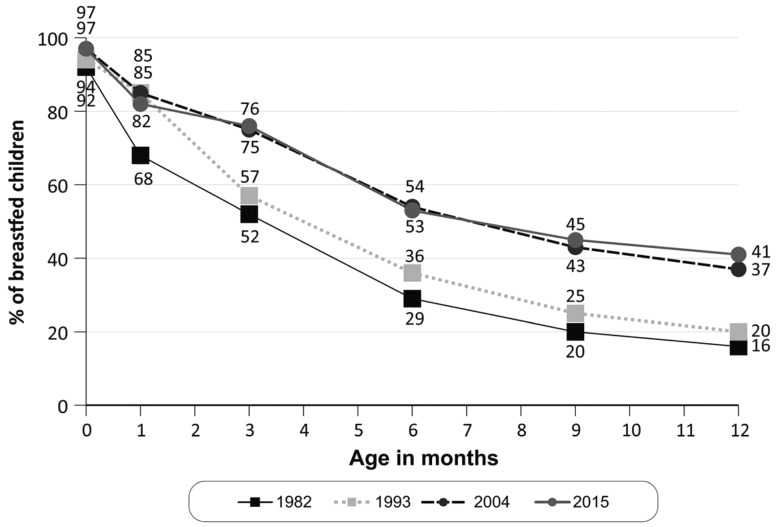
Prevalence of continued breastfeeding at different ages. Pelotas 1982, 1993, 2004 and 2015.

**Figure 2:**
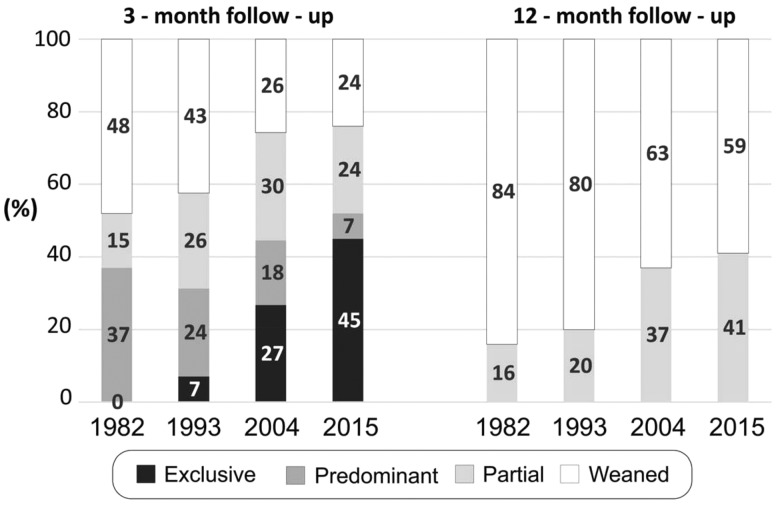
Breastfeeding patterns at ages 3 and 12 months. Pelotas, 1982, 1993, 2004, and 2015.

At all ages, higher proportions of infants were breastfed in 2015 than in 1982 (Figure 1). Figure 2 shows breastfeeding patterns at 3 and 12 months of age. The improvement in prevalence of exclusive breastfeeding at 3 months was most evident. Data are not presented for 1982 because information on feeding child water or tea intake was not collected; the prevalence of either exclusive or predominant breastfeeding in this cohort was 37%, but it is safe to assume that the prevalence of exclusive breastfeeding was virtually zero as the standard practice was to feed children with water and tea from the first weeks of life. For the subsequent cohorts, prevalence of exclusive breastfeeding increased from 7% in 1993 to 27% in 2004 and 45% in 2015, corresponding to an increase of 67% in the prevalence of this practice in the most recent 11-year period. Partial breastfeeding remained relatively stable, reaching around 25% of all infants since 1993. There was a marked reduction in the proportion of fully weaned infants at 3 months of age, with the proportion of children receiving some form of breastfeeding in 1982, 1993, 200 and 2015 being 52%, 57%, 74% and 76%, respectively (*P* for linear trend <0.001). The prevalences of exclusive breastfeeding at 6 months were 0.5% in 1993, 6.5% in 2004 and 14.5% in 2015.

At 12 months, the prevalence of continued breastfeeding increased from 16% in 1982 to 20%, 37% and 41%, respectively, in 1993, 2004 and 2015 (Figure 2) (*P* for linear trend <0.001). The median durations of breastfeeding increased from 3.0 months in 1982 and 1993 to 6.0 and 6.9 months in 2004 and 2015, respectively. In the most recent cohort, few infants were fully weaned between 7 and 12 months, so that in spite of the median value of 6.9 months, the prevalence of breastfeeding at 12 months was 41%.

Table 1 shows the results for prevalence of exclusive breastfeeding at 3 months of age. As mentioned, exclusive breastfeeding in the 1982 cohort is assumed to be close to zero and results for that cohort are not presented. The prevalence increased for all income, maternal skin colour and child sex categories over the past two decades (Table 1). In the three cohorts, exclusive breastfeeding was associated with income, reaching more than half (57.2%) of infants from the wealthiest quintile in the 2015 cohort. Differences according to maternal skin colour or sex of the child were not marked.

**Table 1.** Prevalence of exclusive breastfeeding at 3 months according to family income, maternal skin colour and sex of the child. Pelotas, 1993, 2004, and 2015

Variables	Exclusive breastfeeding at 3 months of age,^a^*n* (range)		
	1993	2004	2015
Family income (quintiles)	*P* = 0.025*	*P* <0.001*	*P* <0.001*
1st (poorest)	3.7	18.4	34.6
	(1.9; 6.9)	(15.8; 21.1)	(31.3; 37.9)
2nd	6.0	22.3	42.8
	(3.8; 9.3)	(19.4; 25.1)	(39.4; 46.2)
3rd	8.1	27.3	42.9
	(5.1; 12.6)	(24.2; 30.4)	(39.5; 46.3)
4th	8.1	30.0	45.7
	(5.1; 12.6)	(26.9; 33.1)	(42.3; 49.1)
5th (wealthiest)	11.4	34.9	57.2
	(7.8; 16.3)	(31.6; 38.1)	(53.8; 60.6)
Maternal skin colour	*P* = 0.731**	*P* = 0.510**	*P* = 0.055**
White	7.6	27.0	45.8
	(6.1; 9.5)	(25.4; 28.6)	(44.0; 47.6)
Brown	5.8	26.2	40.6
	(2.0; 15.5)	(21.0; 31.3)	(36.4; 44.7)
Black	6.3	25.0	43.0
	(3.7; 10.5)	(21.9; 27.9)	(39.1; 46.9)
Child's sex	*P* = 0.922**	*P* = 0.144**	*P* = 0.020**
Male	7.4	25.6	42.9
	(5.5; 9.8)	(23.7; 27.4)	(40.7; 45.0)
Female	7.2	27.6	46.5
	(5.4; 9.5)	(25.6; 29.5)	(44.3; 48.7)
All	7.3	26.5	44.7
	(5.8; 8.8)	(25.2; 27.9)	(43.2; 46.2)

a For trends over time, all *P*-values are <0.001;

* *x*^2^ test for trend;

** *x*^2^ test.

For exclusive breastfeeding at 3 months of age the slope index (a measure of absolute inequalities) increased from 7.4% points in 1993 to 23.2 in 2015 (Table 2). In contrast, the concentration index (an indicator for relative inequalities) fell from 15.9 in 1993 to 8.9 in 2015, indicating that this practice is still more concentrated among the wealthiest, and that although absolute inequalities increased over time, relative inequalities fell.

**Table 2.** Slope index of inequality (SII) and concentration index (CIX) in prevalence of exclusive breastfeeding at 3 and 12 months, according to family income in quintiles

	Cohort	Slope index (SE)		Concentration	
		(%)		index (SE)	
	1993	7.4	(2.40)	15.9	(5.60)
Exclusive breastfeeding					
at 3 months	2004	19.8	(2.30)	12.6	(1.50)
	2015	23.2	(2.60)	8.9	(1.00)
	1982	−9.7	(1.70)	−10.1	(1.80)
Breastfeeding at 12 months					
	1993	−7.5	(3.50)	−6.9	(3.20)
	2004	−4.7	(2.60)	−1.8	(1.20)
	2015	1.2	(2.70)	0.2	(1.10)

SE, standard error.

The increases in prevalence of continued breastfeeding at 12 months of age were observed in all income, maternal skin colour and child sex categories over the three decades (Table 3). For instance, among infants from the poorest families, the prevalence increased from 19.2% in 1982 to 40.5%, in 2015 (*P* for linear trend <0.001) (Table 3). Statistical evidence of inverse associations with income was present in 1982 (*P* <0.001) and in 2004 (*P* = 0.018), but over time the social gradient disappeared. In all four cohorts, prevalence of continued breastfeeding at 12 months was consistently lower among white compared with black mothers. Lastly, girls were more likely to be breastfed than boys, with differences of up to 7.1% points in 1993.

**Table 3.** Prevalence of continued breastfeeding at 12 months according to family income, maternal skin colour and sex of the child. Pelotas 1982, 1993, 2004, and 2015

Variables	Breastfeeding at 12 months %^a^**(range)**			
	1982	1993	2004	2015
Family income	*P* <0.001*	*P* = 0.365*	*P* = 0.018*	*P* = 0.400*
(quintiles)				
1st (poorest)	19.2	21.9	36.3	40.5
	(16.8; 21.7)	(17.1; 27.6)	(32.9; 39.7)	(37.0; 43.9)
2nd	20.0	21.3	40.2	43.0
	(17.6; 22.4)	(16.9; 26.5)	(36.7; 43.6)	(39.6; 46.4)
3rd	13.9	21.1	39.1	39.1
	(11.9; 16.0)	(16.2; 27.1)	(35.7; 42.6)	(35.7; 42.5)
4th	12.8	19.0	38.1	40.3
	(10.8; 14.8)	(14.3; 24.8)	(44.8; 41.5)	(37.0; 43.7)
5th (wealthiest)	12.9	15.2	32.5	43.0
	(10.8; 14.9)	(11.0; 20.6)	(29.2; 35.8)	(39.6; 46.5)
Maternal skin colour	*P* <0.001**	*P* <0.001**	*P* <0.001**	*P* <0.001**
White	13.7	18.3	34.8	39.5
	(12.7; 14.7)	(15.9; 20.9)	(33.1; 36.6)	(37.7; 41.3)
Brown		13.4	37.6	41.8
	24.8	(6.8; 24.7)	(31.8; 43.4)	(37.6; 46.1)
Black	(22.0; 27.5)	29.4	46.2	48.8
		(23.5; 36.1)	(42.7; 49.7)	(44.8; 52.8)
Child's sex	*P* = 0.053**	*P* = 0.002**	*P* = 0.020**	*P* = 0 .012**
Male	14.8	16.3	35.5	39.3
	(13.4; 16.1)	(13.6; 19.6)	(33.4; 37.6)	(37.2; 41.4)
Female	16.7	23.4	39.1	43.2
	(15.3; 18.1)	(20.2; 26.9)	(36.9; 41.3)	(41.0; 45.4)
All	15.7	19.9	37.3	41.2
	(14.7; 16.7)	(17.6; 22.1)	(35.7; 38.8)	(39.7; 42.7)

a For trends over time, all *P-*values are <0.001;

* *x*^2^ test for trend;

** *x*^2^ test.

The slope index of inequality for continued breastfeeding at 12 months fell from −9.7% points in 1982 to 1.2 in 2015 (Table 2). The concentration index changed from −10.1 in 1982 to 0.2 in 2015. Both indices show that in the earlier cohorts, continued breastfeeding at 12 months was more concentrated among the poor and that the inequalities between the poorest and the wealthiest children have been completely eliminated over time.

## Discussion

We documented important improvements in breastfeeding practices over a 33-year period. Exclusive breastfeeding was rarely practised in 1982, but between 1993 and 2004 there was a 4-fold increase in its prevalence at 3 months of age, and between 2004 and 2015 the increase was 67%. Results from national surveys (1986, 1996, 2006 and 2013) confirmed Brazil’s upward trends for exclusive breastfeeding in infants under 6 months of age and continued breastfeeding at 12 months, with the main increases observed between 1986 and 2006 (from 4.7% to 37.1% and from 25.5% to 47.4%, respectively), followed by relative stabilization in 2013 (36.6% and 45.4%, respectively).^15^ The 41% prevalence of continued breastfeeding at 12 months in our 2015 cohort was similar to the 45% prevalence observed in the most recent national survey (2013). The results for exclusive breastfeeding are not comparable: we report a prevalence of 45% at the age of 3 months, whereas the national prevalence for all children aged less than 5 months in Brazil was 37%. Although Brazil experienced a recent deceleration of the gains that were observed between 1986 and 2006,^15^ Pelotas continues to show an ascending trend in both exclusive and continued breastfeeding.

Brazil is internationally recognized as an exemplar country in the promotion, protection and support for breastfeeding.^16^^,^^17^ Such success is the result of a series of actions carried out in the country since the establishment in the 1980s of the National Breastfeeding Program: regulation of the commercialization of infant formula and foods, introduction of the Baby Friendly Hospitals Initiative, creation of the Brazilian Network of Human Milk Banks, adoption of kangaroo mother care as public policy and implementation of the ‘Feed and Breastfeed Brazil Strategy’ (to promote breastfeeding and healthy complementary feeding within the universal primary health care system). These initiatives were scaled up along with media campaigns and major social mobilization events such as the World Breastfeeding Weeks and the World Human Milk Donation Days.

The Pelotas cohort studies were taking place as these initiatives were rolled out. In addition, research on the benefits of breastfeeding has been carried out in Pelotas since the 1980s^18^ and the city hosted one of the participating centres in the WHO Multicentre Growth Reference Study, conducted between 1997 and 1998, which entailed the training of health professionals from the municipality public health system and provided strong support to breastfeeding delivered at households by trained nurses.^19^ The increases in breastfeeding rates between 1982 and 2015 in Pelotas in part reflect the impact of this wide range of initiatives at national and local levels. This may explain why Pelotas has had a better recent track record than the rest of the country.

In terms of socioeconomic inequalities, exclusive breastfeeding was picked up by mothers in the wealthiest quintiles more rapidly than by poor mothers, and by 2015 a difference of more than 20% points had been established. This is in accordance with the inverse equity hypothesis which states that the better-off are in general the first to benefit from newly introduced interventions, due to greater access to information and quality health care.^20^ The finding of an increase in absolute inequalities, expressed as a difference between rich and poor, concomitant with a decrease in relative inequalities, expressed as a ratio, is not unusual when the baseline levels among the poor were very low—only 3.7% in 1982.

It is safe to assume that in the far past, breastfeeding beyond 12 months was universal in all social groups, including the better-off who may have relied on wet nurses.^21^ By 1982, continued breastfeeding was very low in the Pelotas population as a whole, but more common (19%) among the poorest than in the richest quintile (13%). This is likely a consequence of the fact that rich mothers were more likely to use formula or other types of milk than poor mothers in the middle of the 20th century, having adopted artificial feeding to a greater extent due to both marketing pressures and economic advantage. However, as the benefits of breastfeeding started to be disseminated, socioeconomic differences have virtually disappeared by 2015 due to an increase of 30% points in the richest quintile, accompanied by a smaller increase of about 20% points among mothers in the poorest quintile.

Continued breastfeeding was more common among brown and black women compared with white women, but the prevalence of exclusive breastfeeding at 3 months was similar in all groups. These results are consistent with those from a cross-sectional nationwide study conducted in 2013.^22^ Some authors have argued that this finding reflects the historical practice of wet nursing, a social role played originally by slave African women who fed the infants of rich white mothers, a practice that persisted well into the 20th century^21^ although it seems possible that other factors are involved as well. It is interesting to note that even though black mothers tend to be poorer than white mothers, in 2015 they had a near 10%-point lead in terms of breastfeeding at 12 months, whereas prevalence was similar in the five income quintiles.

Our results on longer duration of any breastfeeding among girls in all four cohorts, and of longer duration of exclusive breastfeeding also among girls in 2015, are consistent with previous studies from Brazil and Latin America.^23–25^ Some authors have suggested that parents are more likely to think that sons have greater nutritional needs than daughters, and therefore need to receive formula and other foodstuffs at an earlier age.

The main limitation of this study is that breastfeeding information was gathered from maternal report, being subject to information bias. There are also some differences in the methodologies used to collect the breastfeeding data in the earlier cohorts. In 1982, information on breastfeeding was collected from one-third of the cohort at around 12 months of age, and the other two-thirds at around 20 months; in 1993, information was collected at 6 and 12 months. In both cohorts, mothers provided retrospective information about feeding patterns at 3 months, and the two-thirds of the 1982 cohort who were only seen at 20 months also provided retrospective information about feeding at 12 months. In 2004 and 2015, information was collected cross-sectionally at 3 and 12 months, so that there was no need for recall. It is, nevertheless, reassuring that a recent study conducted with a sample of low-income Brazilian mothers showed a strong concordance between direct observation of breastfeeding, at every 2 months during the first 2 years of life, and maternal report when children were 6 years old (intra-class correlation coefficient = 0.923; *P* = 0.001).^26^ Another limitation is the fact that the 2004 and 2015 cohorts included visits to the all children at 12 months, whereas subsamples were visited in 1982 and 1993; nevertheless, even these samples included over 1300 children.

One should also note that the standard international indicator for exclusive breastfeeding includes in the denominator all children aged less than 6 months; this information is collected through surveys which include children with different ages.^27^ In a cohort that is visited at specific ages, it is not possible to calculate such an indicator.

Another limitation of this study is the lack of information on water and tea intake in the 1982 cohort. At that time, there were no international recommendations regarding exclusive breastfeeding, which were only issued in the 1990s.^28^ The senior authors of the present article (F.C.B. and C.G.V.) were practising physicians in Pelotas in the 1980s and attest to the fact that exclusive breastfeeding was virtually null at the time of the first cohort.

The use of data from four prospective population-based birth cohorts, with low attrition rates, represents a strength of this study. Additionally, all four cohorts were designed in a standardized manner by the same group of researchers, making the four cohorts comparable.

Our analyses confirmed that the positive trends in breastfeeding practices, which were documented for the 1982 to 2004 period,^10^ persisted until 2015. However, less than half of the children in the most recent cohort were being exclusively breastfed at 3 months and received continued breastfeeding on their first birthday. In spite of the progress, the breastfeeding indicators in Pelotas are still far from the ideal. According to the World Health Organization criteria, the breastfeeding duration in the Pelotas 2015 cohort would be classified as ‘poor’ (median ≤17 months) and exclusive breastfeeding practices would be classified at best as ‘fair’ (12–49%).^27^ Of even greater concern is that the beneficial breastfeeding practices are being more rapidly adopted by high-income than low-income women. Action is needed to speed up the improvement of appropriate breastfeeding practices, particularly among poor families which will lag behind if current trends persist.

## Funding

The four cohorts received funding from the following agencies: Wellcome Trust, International Development Research Center, World Health Organization, Overseas Development Administration of the United Kingdom, European Union, Brazilian National Support Program for Centers of Excellence (PRONEX), Brazilian National Council for Scientific and Technological Development (CNPq), Science and Technology Department (DECIT) of the Brazilian Ministry of Health, Research Support Foundation of the State of Rio Grande do Sul (FAPERGS), Brazilian Pastorate of the Child and Brazilian Association for Collective Health (ABRASCO).

## Pelotas Cohorts Study Group

Aluisio J D Barros,^1^ Alicia Matijasevich,^2^ Andrea Dâmaso Bertoldi,^1^ Fernando C Wehrmeister,^1^ Helen Gonçalves,^1^ Joseph Murray,^1^ Maria Cecilia F Assunção,^1^ Mariangela F Silveira,^1^ Marlos Rodrigues Domingues^1^ and Pedro R C Hallal.^1^

^1^Federal University of Pelotas, Brazil and ^2^University of São Paulo, Brazil.

**Conflict of interest:** None declared.

## Supplementary Material

Supplementary Table
