# Supplementary material for: Breastfeeding exclusivity and duration: trends and inequalities in four population-based birth cohorts in Pelotas, Brazil, 1982–2015
Source: Int J Epidemiol. 2019 Mar 18;48(Suppl 1):i72–9. doi: 10.1093/ije/dyy159 (PMC6422059; doi:10.1093/ije/dyy159)
Supplement: Supplementary Table [file dyy159_supplementary_table.docx]

| **Supplementary Table.** Response rates, numbers of children followed-up and timing of each follow-up providing data on breastfeeding during the first year of life in the Pelotas birth cohorts. | | | | |
| --- | --- | --- | --- | --- |
| **Cohort** | **Breastfeeding pattern at 3 months** | **% follow up**  **and sample size** | **Breastfeeding pattern at 12 months** | **% follow up and sample size** |
|  |  |  |  |  |
| 1982 | 12-month  and  20-month interviews | 79.3%  N= 1523  and 87.2%  N= 4934 | 12-month  and  20-month interviews | 79.3%  N= 1523  and 87.2%  N= 4934 |
|  |  |  |  |  |
| 1993 | 6-month interview | 96.8%,  N= 1414 | 12-month interview | 93.4%  N= 1479 |
|  |  |  |  |  |
| 2004 | 3-month interview | 95.7%  N= 4051 | 12-month interview | 94.2%  N= 3989 |
|  |  |  |  |  |
| 2015 | 3-month interview | 97.2%  N= 4156 | 12-month interview | 95.4%  N= 4077 |
